# Safety comparison of four types of rabies vaccines in patients with WHO category II animal exposure: An observation based on different age groups

**DOI:** 10.1097/MD.0000000000005049

**Published:** 2016-11-28

**Authors:** Jun Peng, Sha Lu, Zhenggang Zhu, Man Zhang, Quan Hu, Yuan Fang

**Affiliations:** a Department of Neurology, Union Hospital, Tongji Medical College, Huazhong University of Science and Technology; b Wuhan Centers for Disease Control and Prevention, Wuhan, China.

**Keywords:** age, rabies vaccines, safety

## Abstract

To evaluate the safeties of 4 types of rabies vaccines for patients with WHO category II animal exposure, especially in different age groups.

A total of 4000 patients with WHO category II animal exposure were randomly divided into 4 vaccine groups, and were respectively given with Vaccines A, B, C, and D. And subjects in each vaccine group were divided into 4 age groups (≤5, 5–18, 19–60, and ≥60-year-old groups). Then adverse events (including local and systemic ones) were recorded and compared. Consequently, except for Vaccine B, patients under the age of 5 in Groups A, C, and D suffered from more adverse reactions than those in other age groups. Furthermore, for the children aged less than 5 years, incidence of adverse events following administration of Vaccine B, with the dose of 0.5 mL and production of bioreactor systems, was significantly lower than Vaccines A and D.

Our data showed that rabies vaccines with smaller doses and more advanced processing techniques are of relatively high safety for the patients, especially for the young children.

## 1 Introduction

There are approximately 55,000 deaths of rabies per year in the world, 84% of which occur in rural areas of developing countries.^[1]^ In China, the largest developing country, rabies remains a major public health problem. Because of very high case-fatality ratio (nearly 100%) of rabies, prophylaxis by vaccination is the only protection against rabies, including preexposure and postexposure prophylaxis (PEP). PEP consists of wound cleaning, rabies vaccination, and passive immunization with rabies immune globulin,^[2]^ of which the most important treatment is rabies vaccination. With nerve-tissue vaccines replaced with cell-culture-derived vaccines (CCVs),^[3]^ adverse reactions induced by rabies vaccines have been immensely reduced. To date, a great number of CCVs are available worldwide, including mainly human diploid cell vaccine (HDCV),^[4]^ purified chick embryo cell vaccine (PCECV), and purified Vero cell vaccine (PVRV). Undoubtedly, the 2 latter vaccines are widely used in developing countries.^[3]^ All of them are recommended by the World Health Organization (WHO) due to their high safety and immunogenicity. Several schedules of rabies vaccination approved by WHO have shown to be immunogenic, including the 5-dose Essen regimen and the 4-dose Zagreb regimen (via the intramuscular route), and the Thai Red Cross 2-site regimen (via the intradermal route).^[5]^ Nevertheless, there are still numerous adverse events (AEs) following rabies vaccination reported. Moreover, incidence and severity of people's AEs at different ages vary even if administrated with the same vaccine. Hence, it is imperative for human to assess accurately safety of various rabies vaccines for people at different ages. Here, we compared the safety of 4 types of rabies vaccines for people at different ages who received PEP after WHO category II animal exposure.

## 2 Methods

### 2.1 Ethics

All experimental conditions conformed to the Declaration of Helsinki, and all participants or their legal guardians signed an informed consent form. The protocol of this study was approved by the Institutional Review Board of Wuhan Centers for Disease Control and Prevention (WHCDC).

### 2.2 Subjects

All subjects visiting the clinic of WHCDC from June 2011 to June 2012 were involved in the present study and professionally evaluated as WHO category II exposure to suspect or proven rabid animals according to WHO criteria for animal exposure. All patients lived in Wuhan for more than 6 months, and visited the clinic within 24 hours after exposure. Inclusion and exclusion criteria were defined in the following frame.

Inclusion criteria

1. Female and male subjects over 2 years old
2. WHO category II exposure to suspect or proven rabid animals
3. Not previously vaccinated with antirabies vaccines or other vaccines
4. Not having any acute or chronic disease
5. Normal body temperature
6. To comply with the requirements of clinic trial and participate in follow-up

Exclusion criteria

1. Hypersensitivity to any vaccine component
2. History of thrombocytopenia or other coagulation disorders
3. Abnormal labor birth, asphyxia history, or suffering from congenital malformations, developmental disorders or severe chronic disease
4. Having severe cardiovascular disease
5. Administrated with immune globulin in recent three months
6. Serious adverse reactions following any vaccination before
7. Any contraindication listed in vaccines instructions.

### 2.3 Vaccines

The 4 types of rabies vaccines were mainly different in cell types of culturing, dosages, physical formations, and techniques of production processing, which are described in Table 1.

**Table 1:**
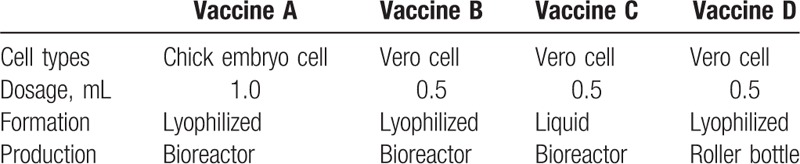
Properties of 4 different rabies vaccines.

### 2.4 Study design

The sample size estimation was conducted according to the “Practical Manual of Sample Size Determination in Health studies” as described previously,^[6]^ a minimal of 864 cases in each vaccine-group was required. A total of 4000 patients visiting the clinic were enrolled and divided randomly into Groups A, B, C, and D, who were respectively given with the 4 different types of rabies vaccines above. And the subjects in each group (n = 1000) were further divided into 4 subgroups according to their ages (≤5, 5–18, 19–60, and ≥60 years old). Vaccines were alternately injected at 1 deltoid muscle under Essen regimen.^[7]^

### 2.5 Safety monitoring

All solicited and unsolicited adverse events were observed and recorded during the clinical trial according to the International Committee for Harmonization Guideline for Clinical Safety Data Management: Definitions and Standards for Expedited Reporting. All subjects were observed within 30 minutes after each vaccination (D0, D3, D7, D14, and D28) for immediate reactions. Particularly, AEs were collected for 72 hours following the first injection of rabies vaccines through telephone interview. The onset and the duration of AEs were recorded on adverse reaction cards (ARCs) by subjects receiving the subsequent 4 injections. Both systemic reactions (fever, allergy, debilitation, dysphoria, nausea/vomitting, diarrhea) and local reactions (pain, erythema, swelling, induration, haphalgesia) were recorded. Then, AEs were classified by the center investigators as related or unrelated to the study vaccines. Adverse reactions were rated according to the Adverse Reaction Rating Scale Guideline for Preventative Vaccine Clinical Trials,^[8]^ which was derived from the pediatric toxicity table of US, National Institutes of Health Microbiology and Infectious Disease Division of Microbiology and Infectious Diseases.^[9]^

### 2.6 Statistical analysis

GraphPad Software was used for statistical analysis, and a *P* value of <0.05 was considered as statistically significant. Categorical variables were tested with *χ*^2^ of the Fisher exact test.

## 3 Results

### 3.1 Subjects and demographics

**Figure 1:**
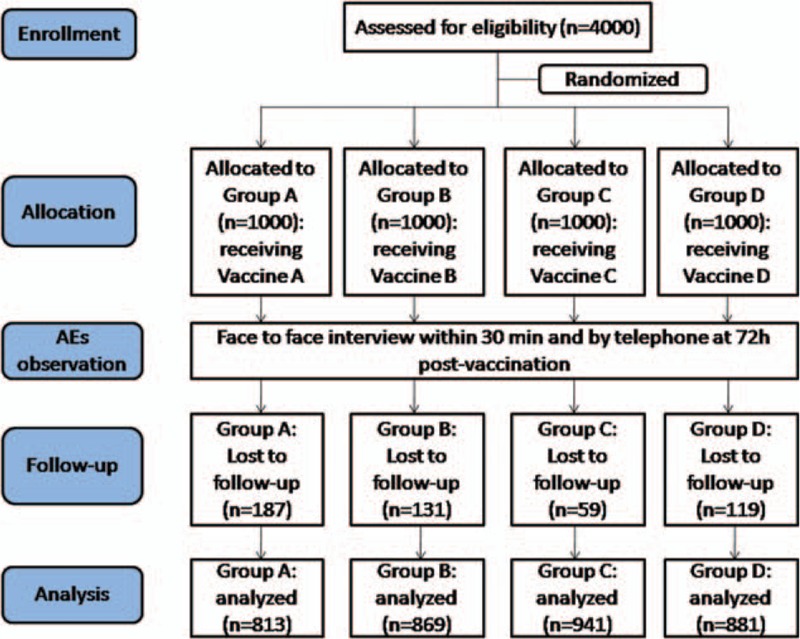
Participant flow.

Three thousand five hundred four of 4000 subjects (87.6%) completed successfully the study, which consisted of 1632 males and 1872 females. Simultaneously, 496 subjects were lost to follow-up for various reasons, including not all of 5 times of vaccination being performed in our clinic (N = 138), telephone follow-up being failed (N = 269), and subjects being in exclusion criteria (N = 89). Yet, no statistically significant differences were observed for lost population between vaccine groups, and between age groups in each vaccine group (seen in Fig. 1).

### 3.2 Children under the age of 5 suffered from more adverse reactions following the rabies vaccination than other 3 age groups

**Figure 2:**
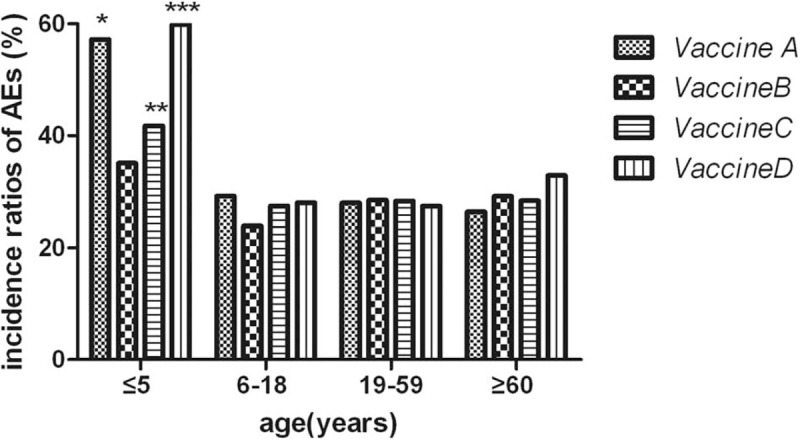
Comparison of AEs among 4 different age groups of each vaccine group. “^∗^” means in Group A: compared with other 3 subgroups (6–18, 19–59, and ≥60-year-old groups), incidence ratio of AEs in ≤5-year-old group was significantly increased (all of 3 *P* values <0.001). “^∗∗^” means in Group C: compared with other 3 subgroups (6–18, 19–59, and ≥60-year-old groups), incidence ratio of AEs in ≤5-year-old group was significantly increased (*P* <0.001, = 0.002, = 0.004 respectively). “^∗∗∗^” means in Group D: compared with other 3 subgroups (6–18, 19–59, and ≥60-year-old groups), incidence ratio of AEs in ≤5-year-old group was significantly increased (all of 3 *P* values <0.001). AEs = adverse events.

We made comparison of AEs among 4 different age groups in each vaccine group and found that incidence of AEs in children under the age of 5 were significantly higher than that of subjects over 5 years old in Groups A, C, and D. Conversely, there was no significant difference in incidence of AEs among 4 different age groups of Group B (seen in Table 2 and Fig. 2).

**Table 2:**
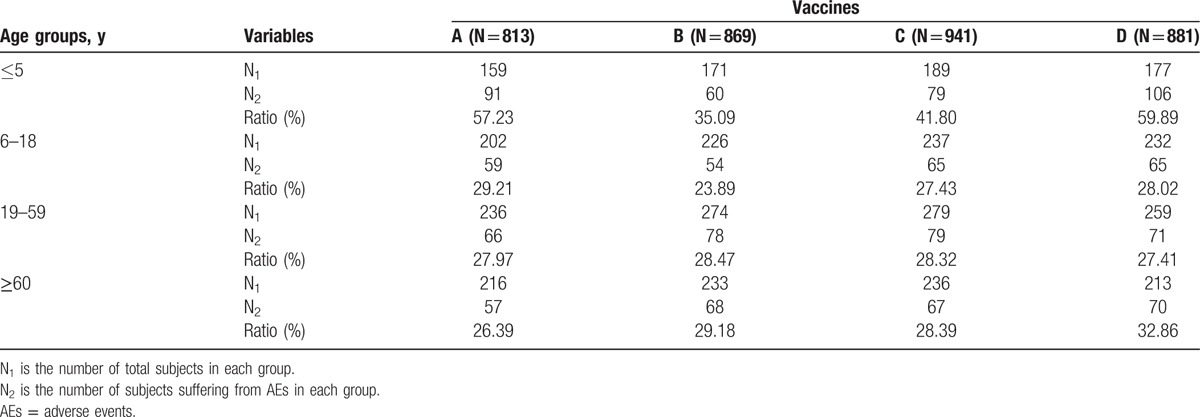
Comparison of AEs among 4 different age groups of each vaccine group.

### 3.3 Vaccine B induced much less adverse reactions of children under the age of 5 following the rabies vaccination than Vaccines A and D

**Figure 3:**
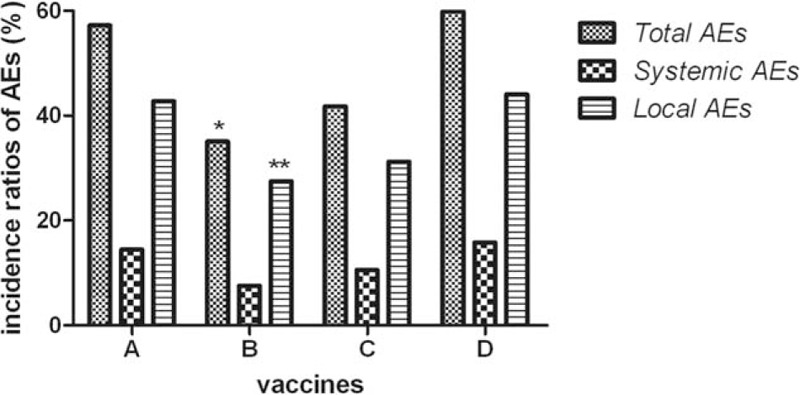
Comparison of AEs of children under the age of 5 among 4 vaccine groups. “^∗^” means that incidence ratio of total AEs in Group B was much lower than that in Groups A and D (both of *P* values <0.001); “^∗∗^” means that incidence ratio of local AEs in Group B was much lower than that in Groups A and D (*P* = 0.004, <0.001 respectively). AEs = adverse events.

**Figure 4:**
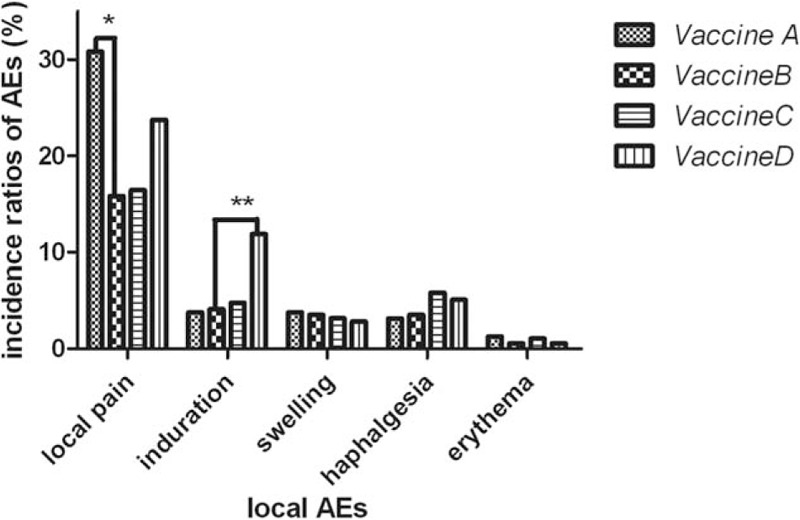
Comparison of various adverse reactions of children under the age of 5 among 4 vaccine groups. “^∗^” means that incidence ratio of local pain in Group B was much lower than that in Group A (*P* = 0.001); “^∗∗^” means that incidence ratio of indurations in Group B was much lower than that in Group D (*P* = 0.008).

Based on the findings above, we focused on adverse reactions of children under the age of 5. As a result, incidence of total AEs in Group B (35.09%) decreased significantly compared with Groups A (57.24%) and D (59.89%). Nevertheless, there was no statistical difference in incidence of Groups B (35.09%) and C (41.80%). Furthermore, systemic and local reactions were respectively recorded and compared. Compared with Groups A (42.77%) and D (44.07%), incidence of local AEs in Group B (27.49%) was significantly decreased, yet which was not statistically different from that in Group C (31.22%). Besides, there was no difference in incidence of systemic AEs among the 4 groups (seen in Table 3 and Fig. 3). More specifically, diverse local and systemic adverse reactions were observed and compared. As for systemic AEs (fever, allergy, etc), there was no significant difference in incidence of any specific adverse reaction among 4 vaccine groups. However, incidence of local pain in Group B (15.79%) was significantly lower than that in Group A (30.82%). Simultaneously, Vaccine B (4.09%) was found to result in much less indurations compared with Vaccine D (11.86%) (seen in Table 4 and Fig. 4).

**Table 3:**
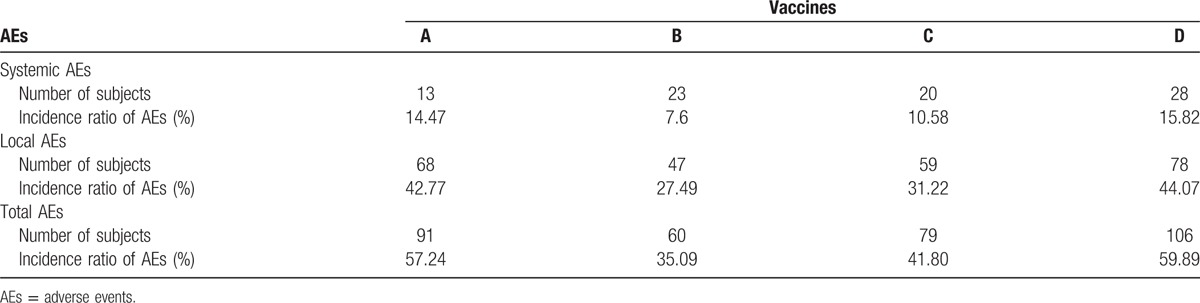
Comparison of AEs of children under the age of 5 among 4 vaccine groups.

**Table 4:**
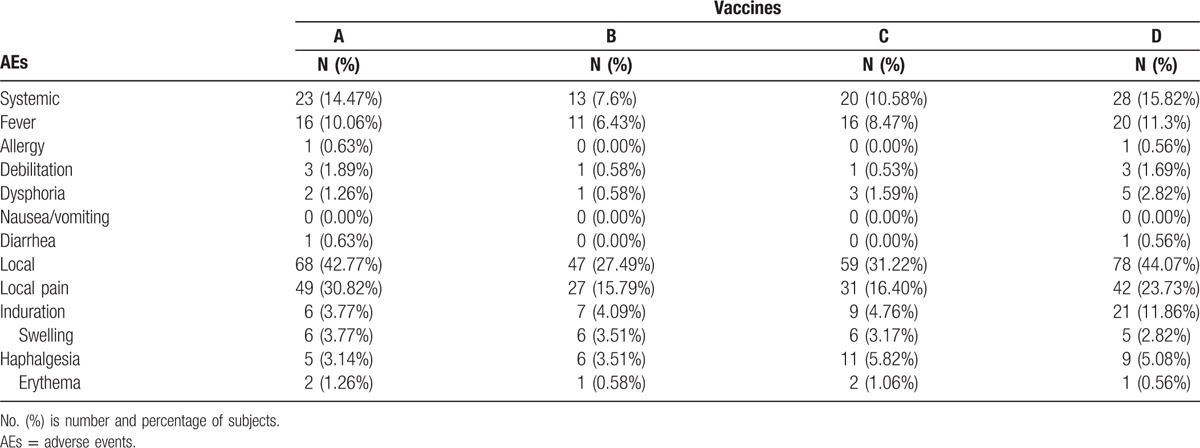
Comparison of various adverse reactions of children under the age of 5 among 4 vaccine groups.

## 4 Discussion

In our present study, we have observed that children (≤5 years old) were more likely to suffer from side effects of Vaccines A, C, and D than the elder subjects. Obviously, age was one of important factors that determined severity of adverse reactions following rabies vaccination. Compared with adults, AEs of children were easily induced due to children's immature immune system, poor tolerance, and emotional stress, which has been observed in our previous studies.^[6,10]^ Interestingly, Vaccine B did not induce more AEs of children under the age of 5 than subjects of 3 other age groups in the present study. Accordingly, Vaccine B seemed to be safer than the other 3 vaccines for children under the age of 5. Then, we compared respectively Vaccine B with Vaccines A, C, and D for children who were ≤5 years old. As a result, Vaccine B led to relatively less AEs than Vaccines A and D, but not Vaccine C. Moreover, Vaccine B was less likely to produce local pain than Vaccine A and indurations than Vaccine D.

As is mentioned above, there are differences in not only culturing cell origination but also dosage between Vaccines A and B. Vaccine B with dose of 0.5 mL was cultured in Vero cell while Vaccine A with a larger dose of 1.0 mL originated from chick embryo cells. To our knowledge, PVRV and PCECV have been approved and widely used in our country for decades. Several comparative studies have confirmed that there is no significant difference in adverse reactions between PVRV and PCECV.^[11,12]^ In this case, we attributed different AEs incidence of the 2 groups to their different doses. That is, children administrated with a relatively small dose rabies vaccine suffered from less adverse events. Therefore, we considered that dosage was one of important factors that affected safety of rabies vaccine, especially for children younger than age 5.

The only difference between Vaccines B and D is the manufacturing technique. Vaccine B was processed in biological fermentation tank while Vaccine D was artificially manufactured in spinner bottle. In early periods, animal vaccines were made by cell cultivation in roller bottle. With the improvement of biological products and technology, application of bioreactor systems was emerging and gradually replacing the traditional process.^[13]^ The numerous advantages of bioreactor mode include simple operation, high volumetric productivity, and low costs.^[14]^ Most importantly, the vaccines produced in bioreactors were much safer than those in roller bottles because of great reduction of residual cell protein, cell DNA, and bovine serum.^[15,16]^ In other words, advanced manufacturing technology of Vaccine B was responsible for the lower incidence of AEs in comparison with Vaccine D. Thus, manufacturing technique was another important factor that had influence in vaccine safety, especially for children younger than age 5.

In addition, Vaccine C in the form of liquid was different from Vaccine B, indicating that formation of vaccines did not affect their safeties.

Nevertheless, there are still some limitations in our study. First, we only assessed adverse reactions of 4 antirabies vaccines in a short time period (within 72 hours); further follow-up studies are needed to determine safety of vaccines. Second, this is just a single-center trial. All the subjects were from WHCDC and Wuhan city in China. Results will be more convincing if the participants were recruited from multiple centers and different cities.

In conclusion, adverse reactions of rabies vaccines were likely to occur on children less than 5 years old. And among the 4 vaccines above, the vaccines that were produced by biological fermentation and administrated with a small dose have comparative high safety on children at risk of rabies.
